# ‘We always find things to learn from.’ Lessons from the implementation of the global maternal sepsis study on research capacity: a qualitative study

**DOI:** 10.1186/s12913-021-06195-9

**Published:** 2021-03-08

**Authors:** Rachidatou Compaoré, Vanessa Brizuela, Anne M. Khisa, Alejandra López Gómez, Adama Baguiya, Mercedes Bonet, Anna Thorson, Evelyn Gitau, Seni Kouanda

**Affiliations:** 1 Research Institute for Health Sciences, Ouagadougou, Burkina Faso; 2 UNDP/UNFPA/ UNICEF/WHO/World Bank Special Programme of Research, Development and Research Training in Human Reproduction (HRP), Department of Sexual and Reproductive Health and Research, World Health Organization, Geneva, Switzerland; 3 African Population and Health Research Centre, Nairobi, Kenya; 4 Programme of Gender, Reproductive Health and Sexuality/Institute of Psychology of Health, School of Psychology, University of the Republic, Montevideo, Uruguay

**Keywords:** Research capacity strengthening, Multi-country study, Maternal sepsis, Maternal infection, Sexual and reproductive health, Qualitative research

## Abstract

**Background:**

Research capacity strengthening could be an indirect outcome of implementing a research project. The objective of this study was to explore the ability of the global maternal sepsis study (GLOSS), implemented in 52 countries, to develop and strengthen sexual and reproductive health research capacity of local participants in low- and middle- income participating countries.

**Methods:**

We carried out a qualitative study employing grounded theory in sixteen countries in Africa and Latin America. We used inductive and deductive methods through a focus group discussion and semi-structured interviews for the emergence of themes. Participants of the focus group discussion (*n* = 8) were GLOSS principal investigators (PIs) in Latin America. Interviewees (*n* = 63) were selected by the country GLOSS PIs in both Africa and Latin America, and included a diverse sample of participants involved in different aspects of study implementation. Eighty-two percent of the participants were health workers. We developed a conceptual framework that took into consideration data obtained from the focus group and refined it based on data from the interviews.

**Results:**

Six themes emerged from the data analysis: recognized need for research capacity, unintended effects of participating in research, perceived ownership and linkage with the research study, being just data collectors, belonging to an institution that supports and fosters research, and presenting study results back to study implementers. Research capacity strengthening needs were consistently highlighted including involvement in protocol development, training and technical support, data analysis, and project management. The need for institutional support for researchers to conduct research was also emphasised.

**Conclusion:**

This study suggests that research capacity strengthening of local researchers was an unintentional outcome of the large multi-country study on maternal sepsis. However, for sustainable research capacity to be built, study coordinators and funders need to deliberately plan for it, addressing needs at both the individual and institutional level.

**Supplementary Information:**

The online version contains supplementary material available at 10.1186/s12913-021-06195-9.

## Background

The World Health Organization (WHO) describes research capacity as “the abilities of individuals, institutions and networks, nationally and internationally, to undertake and disseminate research findings of the highest quality” [1]. Strengthening research capacity at the country level, especially in low- and middle-income countries (LMIC) is critical for the advancement of population health and healthcare [2]. Developing research capacity strengthening (RCS) ensures country ownership of research and research agendas, taking into account country needs, culture, and context in the process of developing studies [3]. It also refers to the empowerment of researchers to define, prioritize problems, and evaluate the best solutions, as well as enhance knowledge translation [4].

Recent reviews of the literature have highlighted the varying definitions of RCS and limited evidence on what works and why [5, 6]. In addition, the challenge of successful implementation of RCS activities needs to overcome several barriers at different levels, from both recipient and provider perspectives [3, 4, 6]. To further compound this, RCS initiatives for sexual and reproductive health and rights (SRHR) are scarce [7, 8].

Evidence shows that participation in a large multi-country study, like the Global Maternal Sepsis Study (GLOSS), [9] can potentially improve clinical management of patients [10] and enhance local research capacity. While GLOSS was not designed to intentionally build the capacities of local researchers, some specific activities (Additional file 1) allowed for capacity strengthening. However, the overall impact on local researchers remains unknown.

This qualitative study aimed to explore if, and how, participation in a large multi-country study – GLOSS - was able to strengthen participating researchers’ capacities. A secondary objective of this analysis was to develop a conceptual framework that could be used to embed RCS for multi-country studies.

## Methodology

This was a qualitative study based on concepts from grounded theory which used a focus group discussion and semi-structured interviews to gather in-depth insights from participants [11]. We have used the Consolidated Criteria for Reporting Qualitative research (COREQ) for qualitative studies for this report [12] (Additional file 2).

### Study setting

Sixteen countries were purposively selected from those participating in GLOSS across three of the study regions: Anglophone Africa, Francophone Africa, and Latin America. The selection of countries within those regions was based on the following criteria: was a GLOSS participating country [9], was a priority country for research capacity strengthening for the UNDP/UNFPA/UNICEF/WHO/World Bank Special Programme of Research, Development and Research Training in Human Reproduction (HRP) [13], had previous experience in multi-country research with HRP, feasibility and ease of travel, interest and availability to participate, and representability for each region in terms of geographic and language diversities.

### Participant selection

Participants in this study were members of local research teams that took part in GLOSS. For the focus group discussion (FGD), we selected a convenience sample of participants who were GLOSS principal investigators (PI) for Latin America. The FGD participants were taking part in a study results workshop in Brazil in 2018. For the interviews we first selected three countries per study region: Anglophone Africa (Kenya, Malawi, and Zimbabwe), Francophone Africa (Benin, Mali, and Senegal) and Latin America (Guatemala, Honduras, and Nicaragua). Then, participants were selected by each country PI and included a diverse sample of interviewees involved in different aspects of study implementation, ranging from data collectors to study coordinators, and from different health facilities, oftentimes located in different country localities. We sought for gender diversity and aimed to interview at least five people per country until we achieved data saturation.

### Data collection and management

#### Focus group discussion

A thematic guide for the FGD was developed based on existing frameworks for evaluation of RCS [3, 14–16]. The following a priori themes based on the existing literature were identified as specific areas for exploration during the FGDs: opportunities and challenges with regards to embedded RCS, experience with research, and institutional support. The FGD was moderated by a senior researcher who had not been part of GLOSS and observed by a member of the GLOSS research team. The FGD was conducted in Spanish and had eight participants. It lasted 2 h, and was audio recorded and transcribed verbatim into Spanish. Additional notes taken by the moderator and observer were used in the interpretation of findings. See Additional file 3 for a copy of the FGD guide.

#### Individual interviews

Preliminary findings from the focus group discussion were used to develop the semi-structured interview guide. All interviewers participated in an online workshop to standardize data collection. Three researchers conducted 63 semi-structured interviews in total, each lasting on average 42 min (range 21–65 min) and completed during site visits to interviewees’ respective work settings. Twenty interviews were in English (8 in Kenya, 6 in Malawi, and 6 in Zimbabwe), 26 in French (9 in Benin, 11 in Mali, and 6 in Senegal), and 17 in Spanish (5 in Guatemala, 5 in Honduras, and 7 in Nicaragua). Sixty-one interviews were conducted face-to-face and two over the phone. The latter were not recorded because of technical difficulties but hand-written notes were used for the analysis. Interviews in Latin America were conducted in December 2018, in Francophone Africa between March and June 2019, and in Anglophone Africa between April and September 2019.

Interviews covered broad questions on participants’ role with GLOSS and prior experience with research, and about opportunities and challenges faced with study implementation and its ability to strengthen research and clinical capacity. Certain socio-demographic data were collected for all focus group participants and interviewees (sex, occupation, place of work) for categorization purposes only. See Additional file 4 for a copy of the interview guide.

#### Trustworthiness and rigour

VB knew all PIs in Latin America participating in the FGD; VB also knew two PIs and two project managers interviewed by her from her role in GLOSS. RC knew all the PIs in Francophone Africa from previous WHO projects (*N* = 3). None of the other interviewees (*N* = 56) were known to any of the interviewers. All interviewers and FGD moderator had experience with qualitative research and in leading these activities. Interviews were conducted individually and confidentially in a private space within participants’ work settings and in the native or the official language spoken in each of the countries (English, French or Spanish) and by each of the interviewers. Interviews were recorded using a digital voice recorder to facilitate analysis after obtaining participants’ permission, and transcribed verbatim.

Excerpts of the transcripts in French and Spanish were translated into English to enable joint team analysis. All transcripts were anonymised to remove identifying information for participants before uploading to a central secure portal for the team to access.

### Data analysis

We used inductive and deductive methods using thematic data analysis starting with *a priori* themes identified from existing literature and refined in a first instance with findings from the FGD and further, at a second stage, with additional emergent themes that arose from the interviews. Examples of emergent themes were: “we were just data collectors” and unintended effects of research. These then informed the final coding used for the analysis of all FGD and interview data. Atlas.ti (version 8.4.18.0 for Windows) was used for the analysis.

To ensure rigour, initial coding and analyses were done by each researcher who led the activity in the respective countries and in the original language after verifying the accuracy of transcripts. Transcripts were coded to identify quotes corresponding to the initial themes in our analysis framework. This coding was then cross-checked by another team member fluent in the language: focus group discussions (ALG and VB), interviews in Spanish (ALG, MB, and VB), interviews in French (MB and RC), and interviews in English (AB and AK), during a validation workshop. A final check was done by four researchers (AB, AK, RC, and VB) to ensure the consistency of the quotes included in this manuscript.

### Public involvement

We did not involve participants directly in this study. However, PIs in each study country helped in organizing site visits, getting needed approvals, and in selecting and contacting participants. Initial findings from this study were shared with all country PIs so that they could extend to the study participants to ensure that everyone agreed with our findings before manuscript submission.

## Results

### Sociodemographic characteristics of participants

A total of 70 participants representing 16 LMICs took part in our study; one participant took part both in the FGD as well as in the interviews. There were no refusals to participate. Fifty-four percent of participants were women and 82% were health workers. Table 1 summarizes overall characteristics of the study participants.

**Table 1:** Characteristics of participants (*N* = 70)

Characteristic	N
Interviewees	
Anglophone Africa	20
Francophone Africa	26
Latin America	17
Focus group participants (Latin America^a^)	8 ^b^
Sex	
Female	38
Male	32
Role in the study	
Principal Investigator (PI)/co-PI	18
Project manager	2
Site coordinator	13
Data entry and management	3
Data collector	32
Data clerk	2
Profession	
Medical doctor	37
Nurse/midwife/clinical officer^c^	20
Statistician	3
Researcher	5
Administrator	5
Type of institution where participant worked	
Public healthcare facility	62
Private non-profit/ Non-governmental organization (NGO)/faith-based healthcare facility	5
Other institution	3
Teaching hospital	12
Experience with research prior to GLOSS	
First time doing research	15
First time in a multi-country study	22

^a^Participating countries in the FGD were: Argentina, Bolivia, Brazil, Colombia, Ecuador, Guatemala, Mexico, and Uruguay

^b^One focus group participant was later interviewed so the total does not add up

^c^Clinical officers are trained and authorized to perform clinical care

### Thematic analysis

Six overarching themes emerged from our analysis and were later used to develop a conceptual framework. These themes, which responded to the data obtained from the FGD and the semi-structured interviews, are described below with supporting data.

### Theme 1: recognizing the need for research capacity

One of our *a priori* themes referred to the exploration of participants’ need for strengthening their own research capacity. This theme included participants’ reports of their own understanding of what research implies as well as their expressed needs regarding RCS as a result of participating in GLOSS. These were linked closely with their past and present experiences with research (including their experience with GLOSS) as well as to specific needs in developing research capacity which emerged during GLOSS study implementation, either at the individual or institutional level. Our findings show that many people across the three regions expressed more interest in research as a result of participating in the study**.** Participants from all regions also identified several RCS needs including i) involvement in protocol development; ii) tailored training and technical support depending on their role in the study and, iii) data analysis and project management. While there were overall commonalities in the expressed needs, these varied across the different participants according to their roles in the study and personal experience with research.

For example, one interviewee reported a newfound understanding of research rigour as a result of participating in GLOSS:

*“Now I saw how hard research is, and that everything needs to be correct and that you need to be vigilant and on top of it, because if not some cases might fall through.”* (Interviewee, Latin America).

Participants also highlighted the importance of having repeat exposure to research, including on different methodologies, as way to strengthen their own research capabilities:

*“To say that we always find in a process things to learn from; for example, the design as it was conceived, the tools as they were used also were for us a factor of experience because we can either adapt them, because we had asked during exchanges with GLOSS, because after this study, can we use the same tools or adapt them for new studies in relation to the same areas.”* (Interviewee, Francophone Africa).

Specific needs regarding data analysis and research management were raised by several of the people interviewed and in the focus group.

*“On the skills of research, I think the main issue, what I would have wanted more was on the data analysis part. Because there was really a good robust data from fifty-three countries. And one of the things that I was really interested in is the skills to say how we really look at this such robust data.”* (Interviewee, Anglophone Africa).

*“We have that difficulty that I was saying that we don't have people dedicated specifically to project management.”* (Focus group participant, Latin America).

Participants mentioned interest in initiating and conducting research locally, and the need for regional collaboration in research:

*“ … in Africa if we can get to a point where we can initiate our own research projects that are funded by us, in which we actually set the research agenda ourselves then that would be good. I think we can achieve that when we’ve got collaboration between the different African countries and support each other so that we end up with the locally driven research agenda”.* (Interviewee, Anglophone Africa).

### Theme 2: unintended effects of participating in research

One of the emerging themes in this study was the unexpected effects of participating in GLOSS, brought about in some settings as well as for some researchers. This theme encompassed positive and negative consequences of research, including changes in clinical practice resulting from participating in GLOSS and how the research protocol was understood by participants. Results showed that the latter was largely understood as a clinical guideline by clinicians across all regions involved in the study, regardless of whether the study was conducted in a private or public facility. Moreover, most of the participants found that GLOSS helped them improve their routine management of maternal infection in their health facilities. For example, participants mentioned that they got to improve their knowledge on maternal sepsis, prevention measures had been improved, as well as clinical management. Some examples supporting this theme included:

*“GLOSS allowed us to see what we were doing well and other things we definitely said, 'this we need to incorporate too’”* (Interviewee, Latin America).

*“ … After the research, the issue of sometimes not having drugs improved. In addition, even some wards, we were going in some wards and they were saying ‘aaah we don’t have the thermometer. Where is the thermometer?’ The thermometers were all bought … to use. Even patient education, I think it improved because by that time I was working in the gynaecological ward where we were admitting those patients with sepsis. The education to the patient, the diet, everything was improved”* (Interviewee, Anglophone Africa).

*“Water stations, washing your hands before and after, now we have them everywhere! In the treatment rooms, at the consultation, it did not exist (before the study), we used to consult like this, without (a water station for hand washing)! Now we have a water station at the antenatal clinic. You can see it! Sterile gloves started to be used... it’s already a change of behaviour*.” (Interviewee, Francophone Africa).

Our study also pointed to limited research experience among some of the health workers, resulting in confusion between their clinical role and GLOSS’ research objectives and implementation.

“*We adapted the protocols to our guidelines … it was well elaborated, with an algorithm. This helped us in how to diagnose and treat*”. (Interviewee, Francophone Africa.)

*“If the project could advocate so that certain (laboratory tests) could be available in referral facilities too, that also is something that could be gained from the project.”* (Interviewee, Francophone Africa.)

Other interviewees also mentioned that participation in GLOSS was an opportunity to create collaboration between clinical health workers and researchers, allowing the former to embrace a research career.

*“In the specific case of the global sepsis study, it was an even bigger challenge ( … ) because we had to reach out not only to the hospital staff that were used to working in research, (and) those large private institutions that had a certain experience in these types of collaborations, but also to smaller hospitals that had never participated in these types of things”* (Focus group participant, Latin America).

Another theme that emerged from the focus group discussion but which was not reported in the individual interviews, related to finding out, through the involvement in the research study, that providers’ had been resistant to change and that the existence of clinical protocols had not yet translated to changes in clinical practice.

*“First of all, we have great diversity in our health facilities, of personnel, where we have to work a bit with resistance to change among the rest of the people. Because we all take for granted that it's ready, that it's been worked on, that there are protocols, that there are clinical guidance documents, that everything is written apparently. And in reality, when we go and are near the people, well, not everyone recognizes the existence of sepsis.”* (Focus group participant, Latin America).

### Theme 3: perceived ownership and linkage with the research study

This theme refers to participants’ feeling of being part of the study and their perception of ownership over the entire process, including the results. This also considers the actions taken to integrate research results into their routine practice and research methods into future investigations. The perceived ownership was reflected in some interviewees’ reports of feeling honoured to be a part of a global project led by WHO; this was also mentioned as a motivating factor for both country coordination team and local field data collectors. Some participants reported being able to see research as the motor that could bring about improvement in their health facilities and clinical practice as a result of participating in GLOSS. For others, the relevance of the research question to their context made them become more involved in the study.

*“In our country, I think the fact that we involved institutions that had never participated in any studies, much less a global study, as you well said, led by WHO. That was an important motivating factor”.* (Focus group participant, Latin America).

*“That such an important study considered my small private health clinic to be part of it made me feel that I was doing something valuable!”* (Interviewee, Francophone Africa).

However, some of the participants identified the fact that they hadn’t been involved from the beginning of the project -e.g., in writing the protocol and developing the data collection tools- as the main difficulty in feeling they were part of the GLOSS research group and in taking responsibility for any difficulties encountered during the implementation of the study.

“ … *I didn’t participate in the (study’s) birth, I didn’t participate in the baptism, I did not participate in the education, but they want to blame me for the bad education of the child! I say no!*” (Interviewee, Francophone Africa).

Relatedly, some participants thought that it was important to engage country team members early in the process to ensure they fully understood the research cycle, from conceptualization of the study to implementation to analysis of results.

*“ … We make sure they are part from the planning stage, when we are planning for the study, planning for the training and make sure they are part and parcel of that. They are actually involved in the actual planning, they take part in the facilitation of the training and also when the data collection comes they are on the ground with the research staff also collecting data. That gives them a sense of a way a collaborative study is done … How to collect data, how data entry is done and then when now we come to the stage where we are allowed to analyse local data then they could also be part and parcels of that”.* (Interviewee, Anglophone Africa).

An issue that came up among participants from Africa had to do with authorship of papers published relating to the study. They brought up how being left out of publications or not being a lead author can limit the feeling of ownership of the study. Contrastingly, none of the interviewees from Latin America brought up this topic.

*“ … a lot of health research is about Africa but researchers in Africa itself usually would not get the credit, so to speak, if you participate in any international study like, for example, the ones that I was mentioning. If I am doing the analysis and the authorship is done, the African researchers would be number seven, number eight on the authorship list, maybe because the funding comes from elsewhere.”* (Interviewee, Anglophone Africa).

### Theme 4: “We were just data collectors”

The theme of power dynamics and potential differentials that might impact the research capacity of country and local teams as well as their experience with research was another important aspect identified with this study. Within this theme, we included the different examples of perceived imbalance in access to knowledge and research capacity strengthening that could influence or undermine research capacity building, particularly with regards to how participants perceived themselves and their role. Many participants from different countries perceived the study implementation as a top-down approach.

“*Because there was no flexibility in making changes to the forms, there was a reaction ‘we should have the option to review this’ instead of it being standardized. This is a management perspective.”* (Focus group participant, Latin America)

*“We mostly played the part of the arms for collecting data, but we had less responsibility during the conception and during the analysis.” (*Interviewee, Francophone Africa).

*“The first difference is that in (my prior experience with a network I belong to) we were the data collectors, but we were also involved in the presentation of the data. Like we went to (the country) as part of the people who were involved in the research team. But in GLOSS we were just the data collectors*.*”* (Interviewee, Anglophone Africa).

Some participants highlighted the fact that international research partners and funders often rely on the same people to lead the research projects in-country. These PIs, in turn, usually replicate the same power dynamics within their own research teams.

*“Here in (our country), we know (the country coordinator) only! ‘Dr. maternal sepsis’! I don't know, they said they were going to send the results, nobody knows, we don't know. Everything, it's (the coordinator)!”* (Interviewee, Francophone Africa).

### Theme 5: belonging to an institution that supports and fosters research

Institutional support has been identified as critical to RCS and this came out clearly in the results of this study. This theme encompassed data relating to environments that encouraged and facilitated research, as well as broader aspects of institutional support including good research practice, research ethics, dedicated time and funding for research, including the existence of national research bodies that foster engagement with research.

Some interviewees reported that enabling environments favour implementation of research.

*“I think maybe because also our environment is very research friendly. It has quite a number of, like we are saying so many international research projects being done and in institutions you will find they are tuned to research. So, whenever a research project comes, the research governing council approves it. You find that problems are very few. People usually like cooperating in that kind of project activities so there were no negative experiences.”* (Interviewee, Anglophone Africa).

On the other hand, participants reported several challenges which restricted the implementation of the research study in their health facilities, such as resource and organizational constraints, including lack of dedicated time for research and financial resources in general. They interpreted these as lack of institutional support for research.

“*We need to manage, but we also need to see patients, perform C-sections, attend births, then, but the time is very limited and very valuable.”* (Interviewee, Latin America).

*“Research must be funded, there must also be incentives, incentives for students and even for providers to get more involved in research.”* (Interviewee, Francophone Africa).

*“If (GLOSS) had come also with a component of funding that allowed incorporation of maybe three or four resident doctors who were training to be specialist, if there was an element of that , such that we say if there are four residents involved in this study, then it would (have) been a very good opportunity for mentoring … ”* (Interviewee, Anglophone Africa).

Others referred to a broader unsupportive research environment as reflected by poor country leadership with regards to public health research.

*“Perhaps the biggest challenge and conflict was obtaining the authorizations. The (national ethics committee) approved us, but the (national body in charge of supervising research in human subjects), which is an entity part of the Ministry of Public Health, a week before the start of the study sent us a letter, I promise you, the letter of recommendation was longer than the protocol … ”* (Interviewee, Latin America).

*“Research has been the last concern of politicians, it is only in recent years, in the last five years that they have begun to take an interest in research in our country … In (my country), I will not hide it, the research has not so far gone to the level of those ( … ) who are in direct contact with the patients, and research has not actually gone down there (to health facilities).”* (Interviewee, Francophone Africa).

### Theme 6: presenting study results back to study implementers

The final stage of the research process, dissemination of results and introduction of any necessary changes, was also identified as a critical component of research capacity. This theme includes all references to providing feedback and dissemination of results and/or other findings to everyone involved in implementing the study. Many participants in Africa complained about the lack or the delay in reporting back the results of the study.

*“We need to know the results of what we had collected. We need to see the way forward. We need the feedback because after collecting data, after doing the research, we need to know what is the way forward, what is the current situation in our country? What were the policy makers saying, is there, and are there any interventions on our study, which we have carried out?”* (Interviewee, Anglophone Africa).

*“We know what is happening in our maternity hospitals, we know what is happening in the boundaries of our country, but we do not know what is happening in other countries. However, I think that in order to try to improve our practices, we need to know what is happening elsewhere. If they perform better than us, what do they do that we don't? What do they practice and we don’t? It allows us to question ourselves and, why not, copy what they do well, to help improve maternal health.”* (Interviewee, Francophone Africa).

In contrast, many participants in Latin America stated that country PIs had organized sessions to present country-specific data obtained from the study, even if the overall results of GLOSS were not yet available. In fact, in Latin America, participants were surprised about the time it took to publish overall results.

*“I imagine you already know that after they presented to those of us who worked on (the study), after the results were presented, that already generated a workshop that we did on sepsis.”* (Interviewee, Latin America)*.*

Some participants referred to issues with translation of knowledge for policy; there were examples of government officials requesting that results were modified or tweaked to avoid releasing data that might reflect on them negatively.

*“We are afraid not only of the publication per se and individually to send to the journal, at the political level they are afraid of knowing the data. Knowing that x, y, z indicators are wrong and because that requires us to confront other countries and the region. We can collect the data and say we have it there but to evidence it is hard not only from a scientific point of view but also political because politics have serious implications for a government, for the state*.” (Focus group participant, Latin America).

### Development of a conceptual framework

**Fig. 1:**
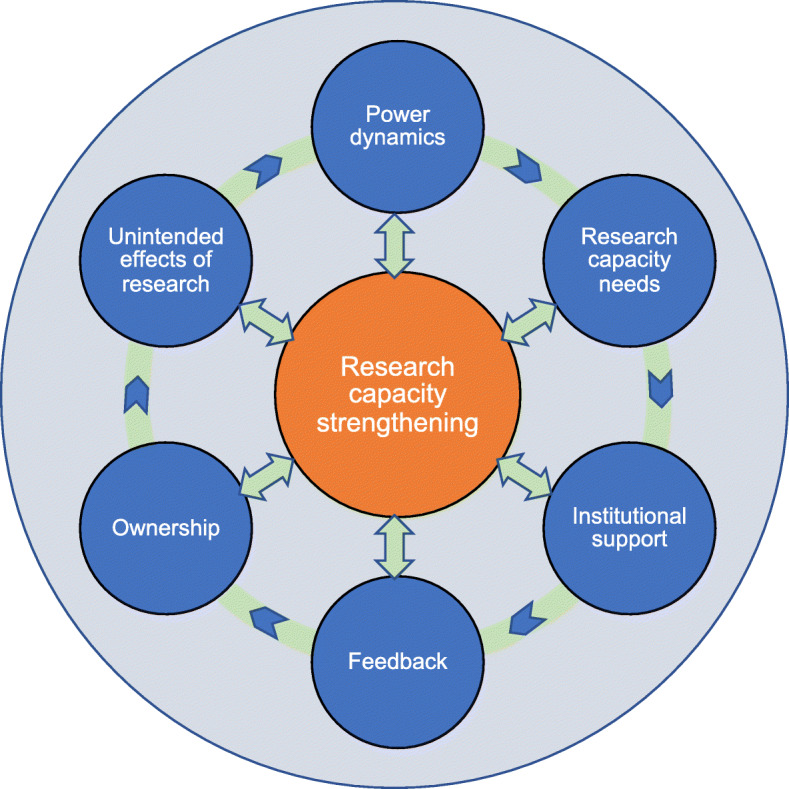
Conceptual framework for research capacity strengthening (RCS)

Using the results of the thematic data analysis, we developed a conceptual framework for embedded RCS in multi-country studies, which aligns with existing evidence and which should be validated for use in further projects. Our framework draws from each of the themes under analysis in this study to result in six broad, overarching concepts. The different concepts were independent but interrelated and all acting within a specific context (Fig. 1).

## Discussion

Our study sought to explore the extent to which participation in a multi-country maternal health study was able to strengthen individual and institutional research capacity. Our findings highlight the need to build on local research capacity, as expressed by most participants regardless of their role and their context. The emerging commonalities in different regions allowed us to develop a conceptual framework that builds on the six overarching themes used in the analysis.

The most frequent theme throughout the interviews and focus group discussion was related to “recognizing the need for research capacity,” where demands for individual and institutional skills building, and for linkages and collaboration between local and regional bodies were made. Needs for RCS in LMICs have been raised before [6, 17]. Our findings stressed the need for specific training in research methodology, based on prior experience with research and the individual’s role in the study, and in project management especially for research coordinators. In a previous study, Ogundahunsi et al. showed that although identifying and supporting individuals with leadership and managerial skills remains a challenge, research project management at the institutional level was important for research capacity growth and sustainability [18].

Another important theme from our analysis referred to unintended effects of research. Participants reported implementing changes in how they managed women with infection and sepsis as a result of their participation in GLOSS. Some of these changes could be expected, to some extent, as GLOSS included a campaign aimed at increasing awareness among healthcare providers working in GLOSS participating facilities [19]. Some studies have shown that the action of conducting research could be an intervention in and of itself, as participation in research can increase awareness on the topic of interest and, therefore, bring about improvements in how patients are cared for [20–22]. However, perhaps due to poor understanding of clinical research, data collected showed that for many participants the GLOSS study protocol was mistaken for a clinical guideline. This is similar to what others have found whereby participants in research studies were more interested when these were “close to practice” [14].

However, the effects of participation in research were not always positive. Involving people with little prior exposure to research can be challenging, as they might not understand the complexity of the whole research process. Our findings underlined how limited experience with research can sometimes hinder the capacity of study implementers in understanding how each aspect of the research processes is integral to the execution of the whole study: data collectors are critical, as are coordinators, project managers, statisticians, and public health specialists.

Moreover, although there is an opportunity for local research teams to positively impact maternal sepsis management, challenges regarding institutional management and resource allocation, may hinder such improvement [10].

Delayed sharing of results of the study was raised through the interviews as one of the weak points of this process. Many participants were convinced that learning about the relevant findings of the study could help improve their clinical practice as well as encourage them to engage in future studies. Cooke [14] suggested that, beyond the common methods of dissemination of research results, capacity building success should also be measured through its impact on practice and even broader, on the health of patients and communities. RCS is not complete if local researchers are not informed of the results and the impact of their participation in the study, especially with regards to findings emerging from their own study sites.

In addition, difficulties or delays in communicating research results to country level participants is a reflection of the interplay of power dynamics between country research teams, officials, and international collaborators [6, 23, 24]. The question of who is implementing the study may have an impact on how results are perceived by local implementers and how they take ownership over them [14, 20]. Another related issue highlighted in our study referred to how first and last authorship in publication of research results is determined, a topic of great current interest in the global health community [6, 14, 18, 25–27].

The lack of institutional support we found in this study, in addition to presenting challenges with regards to implementing research, could also be a barrier to the establishment of some good initiatives arising through study implementation, such as collaboration between partner institutions and facilities, and research centres. However, we are unclear if these collaborations would survive beyond the GLOSS project, which was something that other papers have already pointed to [6]. The existence of an environment that favours research is therefore paramount to strengthening research capacity, something that has already been reported as essential to RCS by others [6, 28].

While RCS was mentioned in the GLOSS study protocol and there were specific actions implemented during the study execution, RCS was not included as a primary or secondary objective [9]. Studies that fail to include explicit RCS objectives have been described as vertical research project strategies [6], whereby RCS activities are focused more on benefitting the successful completion of a short term project with high-quality research outputs, than in developing sustainable research capacity. Of note, there is an initiative led by WHO through the HRP Alliance to help strengthen research partners’ research capacity in SRHR [13].

One of the major strengths of this study is the diversity of the sample which included 70 participants from 16 LMICs in three regions speaking three different languages, allowing for a global perspective and comparison of local teams’ perceptions on RCS. The findings from this study also provide an opportunity for local researchers to assess their own needs in RCS, using lessons learned from this study to better inform the design and implementation of activities aimed at strengthening research capacity in future research collaborations.

However, this study has some limitations. First, since interviews were conducted at the participants’ workplace, these were sometimes interrupted, potentially impacting the openness of the participants and therefore, the quality. Second, one of the researchers in this study played a role in coordinating GLOSS and this involvement could have led to potential response bias. However, most of the interviewees were not aware of her participation in GLOSS. Third, we only conducted one FGD in one region, potentially limiting the themes that guided our initial analysis framework. Nonetheless, the fact that the major themes were elicited consistently throughout the analysis of more than 60 interviews in all nine countries speaks to the comprehensiveness of our analysis which could make up for our decision to have only one FGD.

To advance research in SRHR, researchers in LMICs need continuous and long-term support to conduct studies based on national needs and priorities. They also need the training and exposure to research needed to improve quality. Study research teams need to disseminate research findings to the relevant decision-makers who can, in turn, translate these into evidence-based policies and programs to improve population sexual and reproductive health. It is important to engage local researchers early in the process to ensure the experience is a true research capacity strengthening one. The success of efforts in building research capacity in LMICs will still ultimately depend on the political will for credible, adequately financed, and responsive capacity-building plans based on a thorough situational analysis of the resources needed for health research and the inequities and gaps in health care.

## Conclusion

Participating in health research can increase interest in conducting research, improve research capacity and improve patients’ clinical management. There are important capacity building needs with regards to training and support through involvement in protocol development, study implementation, data analysis, results dissemination and project management, as well as the necessary institutional support. Considerations must be made with regards to how power dynamics, local ownership, and vested interests play a role in multi-country studies. Our findings indicate that an early introduction of equitable authorship guidelines and publication plans may be one way in which to address potential power imbalances. While these power imbalances may shift as a result of unintended RCS during the implementing of research, results of this paper show that more needs to be done to develop sustainable capacity to ensure that LMIC researchers can actively engage in the research process.

## Supplementary Information

**Additional file 1: Appendix A**: RCS strategies implemented during GLOSS.

**Additional file 2: Appendix B** “We always find things to learn from” COREQ (COnsolidated criteria for REporting Qualitative research) Checklist.

**Additional file 3: Appendix C**_Focus group guide.

**Additional file 4: Appendix D**_Interview guide.

## Data Availability

The data that support the findings of this study are available from the corresponding author on reasonable request.

## Abbreviations

COREQ: Consolidated Criteria for Reporting Qualitative research
FGD: Focus group discussion
GLOSS: Global maternal sepsis study
HRP: Human Reproduction Program
LMIC: Low and middle income countries
NGO: Non-governmental organization
PI: Principal investigator
RCS: Research capacity strengthening
SRHR: Sexual and reproductive health research
WHO: World Health Organization
