# Supplementary material for: ‘We always find things to learn from.’ Lessons from the implementation of the global maternal sepsis study on research capacity: a qualitative study
Source: BMC Health Serv Res. 2021 Mar 8;21:208. doi: 10.1186/s12913-021-06195-9 (PMC7938552; doi:10.1186/s12913-021-06195-9)
Supplement: Supplementary file 1 — Additional file 1: Appendix A: RCS strategies implemented during GLOSS. [file 12913_2021_6195_MOESM1_ESM.docx]

**Appendix XX: RCS strategies implemented during GLOSS**

1. *Involvement of doctoral students in study implementation*. Some country PIs designated doctoral students as leaders in different aspects of study implementation. These students were focusing their doctorial theses on maternal sepsis.
2. *Training of country PIs following a train-the-trainer model*. GLOSS coordinating team conducted two trainings with all country PIs and co-PIs, in a training-of-trainers model. The first was a 2-day in-person training on the protocol and data collection tools. The second was a remote, follow-up, half-day training to review issues raised during piloting of the tools and for query clarification.
3. *Provision of training materials for country PIs*. The GLOSS coordinating team provided each country PI with a training packet that included standard presentations for country decision-makers and researchers involved in data collection, in addition to all the data collection tools, a manual of operations, and the full protocol as well as all the materials relating to the awareness campaign that accompanied. All training materials were translated into French, Portuguese, Spanish, and Russian, at the request of participating countries.
4. *Request of input and leadership on additional, secondary analyses*. After data collection was completed all country study teams were asked to provide research questions they wanted to explore from data collected for the study and that could be used for subsequent analyses. Secondary analyses teams were created based on common questions/themes and each team was entrusted with assigning a writing team, a statistician/data analyst, and review team. Special considerations were made for doctoral students and junior researchers to lead on these analyses.
5. *Agreement on authorship for main and subsequent manuscripts*. To ensure open, transparent, and fair authorship in all GLOSS-related publications, an authorship agreement was obtained from all country teams. A GLOSS research group was established including up to three members of each country research team. The authorship agreement included recommendations to use group authorship for main analyses and for all subsequent manuscripts to follow ICMJE recommendations and be published on behalf of the research group.
6. *Development of communication tools for dissemination of results*. Visually attractive infographics and presentations were developed including global and regional results to help disseminate study findings. These materials were also translated into several languages, at the request of participating countries.
