# Supplementary material for: ‘We always find things to learn from.’ Lessons from the implementation of the global maternal sepsis study on research capacity: a qualitative study
Source: BMC Health Serv Res. 2021 Mar 8;21:208. doi: 10.1186/s12913-021-06195-9 (PMC7938552; doi:10.1186/s12913-021-06195-9)
Supplement: Supplementary file 3 — Additional file 3: Appendix C_Focus group guide. [file 12913_2021_6195_MOESM3_ESM.docx]

**GLOSS+. Evaluating GLOSS’ ability to develop and strengthen research capacity**

**Focus group guide**

PREPARATION

- Informed consent for participants

- Flip chart

- Recorder

FOR CONSIDERATION DURING NOTE TAKING

- Identify participants by placement around the room to be able to identify contributions

- Observe group dynamics: who is participating? Who seems uninterested? Who dominates the conversation?

- Observe for parallel conversations or discussions that might arise

OBJECTIVE OF THE FOCUS GROUP

To obtain more information on the general objectives of GLOSS+:

1. To assess country coordinators’ experience with the GLOSS project
2. To assess challenges and opportunities in developing research capacity at the local level
3. To develop recommendations on research capacity strengthening actions

Secondary objectives

1. To document the process of implementing a multi-country study useful for future such experiences
2. Assess country engagement as per their involvement in GLOSS
3. How would you describe your role in GLOSS? What did being a country coordinator mean/imply?
4. What factors facilitated the fulfilment of your role responsibilities? (PROBE: training? Support from WHO HQ? Support from colleagues or regional coordinators?)
5. What factors challenged or acted as barriers for the fulfilment of your role responsibilities?
   1. (optional) How did this role compare to prior experiences you might have had with other research studies?
   2. (optional) How did the implementation of this study differ to any other studies you were involved in? In what ways was it similar?
6. How would you qualify your experience participating in this study? (PROBE: do you feel better equipped to conduct another research project in your setting? Did you learn anything new? Do you think participating in this experience helped you grow professionally? Did you pass any career milestone with help from GLOSS? Have you gained formal or informal merits from leading the country study? Do you think that participating in this experience hindered your everyday work?)
7. Do you feel ownership in GLOSS and related processes? Empowered to develop and integrate GLOSS with own research interests and needs? (PROBE: bottom-up or top-down strategies for study design, implementation, planned analyses and publications, will the study participation provide you with possibilities to spearhead country analyses? Which tools or activities –if any– do you think will be helpful/are missing from the process to support the next steps of analyses and publications writing? Are there more colleagues other than you involved in GLOSS activities and perhaps benefitting from this as positive for their careers?)
8. What would have helped in improving the implementation of GLOSS at the country level? How would you have done this?
   1. If you had to identify three conditions that facilitated the implementation of GLOSS in your country, what would these be?

(Explore political will from decision makers, a culture of evidence-based policy-making, and institutional capacity for national research strengthening (i.e. human and financial resources, an SRH research agenda, access to information, stakeholder interest in research capacity strengthening), capability and expertise of the country coordinator, recognition within their country, leadership, relationship with stakeholders).

- 1. If you had to identify three conditions that challenged the implementation of GLOSS in your country, what would these be?

(Explore political will from decision makers, a culture of evidence-based policy-making, resistance to SRH, institutional capacity for national research strengthening (i.e. human and financial resources, an SRH research agenda, access to information, stakeholder interest in research capacity strengthening), national research institutions, research training.)

1. What would need to be further developed at your institution/in your countries in order to strengthen national capacity in SRH research?
2. If you were to conduct a country-wide research project, what other skills/knowledge do you feel you need? (PBOBE: do you have sufficient human resource capabilities to lead a research study? Do you feel you have the technical skills needed to conduct a research study?)
   1. How would you go about filling any research gaps you may have? (PROBE: approach other colleagues from the GLOSS network, take a course in research methods, etc.).
3. Is there anything else you would like to add?

SUMMARY OF MAIN FINDINGS

After everybody has had a chance to express their thoughts and opinions the moderator will summarize the key points made and obtain consent from the group that these are the main issues raised during the discussion. This will be the time to include any pending or additional thoughts.

**Thanks for participating!**
