# Supplementary material for: ‘We always find things to learn from.’ Lessons from the implementation of the global maternal sepsis study on research capacity: a qualitative study
Source: BMC Health Serv Res. 2021 Mar 8;21:208. doi: 10.1186/s12913-021-06195-9 (PMC7938552; doi:10.1186/s12913-021-06195-9)
Supplement: Supplementary file 4 — Additional file 4: Appendix D_Interview guide. [file 12913_2021_6195_MOESM4_ESM.docx]

**GLOSS+: A process evaluation of a multi-country study in strengthening research capacity**

This interview is part of a study aimed at understanding your experiences with the implementation of the Global Maternal Sepsis Study (GLOSS) that was conducted in 53 countries across the globe, including yours.

This study is being coordinated by the World Health Organization and the HRP Alliance. The goal of this qualitative study is to assess the experience of country coordinators and WHO country staff with the GLOSS project, including challenges and opportunities that arose as a result of participating in the study. Another important objective is to develop recommendations for strengthening maternal health research capacity for global consideration.

**This interview is confidential and its purpose is to gather your thoughts and opinions about the implementation of the Global maternal sepsis study.** This interview should not take longer than 1 hour. There are no right or wrong answers, but rather this is an opportunity to hear your thoughts about the topic as a (COUNTRY COORDINATOR/GLOSS STUDY PROJECT MANAGER/WHO COUNTRY OFFICE STAFF) participating in this study. If you agree to this, I will record the conversation so that I can later remember what we talked about. I will likely be taking notes as well while we speak. I will also ask you to complete and sign an informed consent form. You will keep a signed copy with you.

Thanks in advance for agreeing to participate in this activity.

**To start off, a few questions about yourself …**

1. Sex: (OBSERVE)

|  | Female |  |
| --- | --- | --- |
|  | Male |  |

1. What country are you from? (KNOWN AHEAD OF TIME)
2. What is your professional background? How long have you been working in this field?
3. What is your job title?
4. What was your experience with multi-country studies before participating in GLOSS? What about research in general?
5. [IF YES] What were some of the differences you found in leading this study compared to other studies you were involved in?
6. [IF NO] How would you describe this experience?
7. How would you describe this experience with regards to learning new research skills/doing research? (PROBE: What were these? Did you learn any new research skills or about any new topics in maternal health? What were these? Did your participation in GLOSS spark an interest in research?)
8. How would you describe this experience with regards to implementing change in your setting? (PROBE: did this experience help in changing possibilities for research? Career development? How the issue of maternal sepsis was handled? Were you able to engage with other researchers at your institution, in your region? Did new ideas for collaboration emerge?)
   1. If there were any negative experiences mentioned (PROBE: why do you think this was? what would you have changed to make this a positive experience? Do you think this change could have been brought about differently?)
   2. If there were positive experiences mentioned (PROBE: how did this come about? What made it positive? How did participation in GLOSS help in this specific issue? Do you think this change could have been brought about differently?)
9. What would have helped in improving the implementation of GLOSS at the country level? How would you have done this? What would you have needed to get this done?
10. What changes would you make to the design, implementation and planned analyses and output of GLOSS to ensure professional and institutional growth and strengthening? (PROBE: more training? More mentoring? Support in data collection, management, and analysis? Empowerment? )
    1. Did you feel you were given an opportunity to strengthen your own research capacity (e.g., data collection or data analysis, protocol development, plans for publications or conference presentations)?
11. As you may know, this study was led and coordinated by WHO in Geneva, coordinated at a regional level by the team in [Burkina Faso/Nigeria]. What worked during study implementation regarding the support you received from WHO and/or the regional coordinator? Did you feel supported by WHO and/or the regional coordinator during the study period? (PROBE: did you receive sufficient support? Did their support facilitate/hinder the implementation of the study? Did their support offer new skills for study implementation?)
12. If we were do start over, what things would you keep the same and what things would you change? (PROBE: were the trainings sufficient? Was the support provided by the regional coordinator help? Did you feel that your questions were answered properly?)
13. If you were approached by WHO or other institution to conduct another maternal health study, how would you respond to this and why? (PROBE: do you feel better placed to conduct research in the field? Knowing what you know now, would you start off differently? How?)
14. Is there anything else you would like to add?

**Thanks for participating!**
